# Compression-based distance (CBD): a simple, rapid, and accurate method for microbiota composition comparison

**DOI:** 10.1186/1471-2105-14-136

**Published:** 2013-04-23

**Authors:** Fang Yang, Nicholas Chia, Bryan A White, Lawrence B Schook

**Affiliations:** 1 Division of Nutritional Sciences, University of Illinois at Urbana-Champaign, Urbana, Illinois, USA; 2 Institute for Genomic Biology, University of Illinois at Urbana-Champaign, Urbana, Illinois, USA; 3 Loomis Laboratory of Physics, University of Illinois at Urbana-Champaign, Urbana, Illinois, USA; 4 Department of Animal Sciences, University of Illinois at Urbana-Champaign, Urbana, Illinois, USA; 5 Department of Surgical Research and Health Sciences Research, Mayo Clinic, Rochester, Minnesota, USA

**Keywords:** Microbiota comparison, Microbiome analysis, Compression-based distance

## Abstract

**Background:**

Perturbations in intestinal microbiota composition have been associated with a variety of gastrointestinal tract-related diseases. The alleviation of symptoms has been achieved using treatments that alter the gastrointestinal tract microbiota toward that of healthy individuals. Identifying differences in microbiota composition through the use of 16S rRNA gene hypervariable tag sequencing has profound health implications. Current computational methods for comparing microbial communities are usually based on multiple alignments and phylogenetic inference, making them time consuming and requiring exceptional expertise and computational resources. As sequencing data rapidly grows in size, simpler analysis methods are needed to meet the growing computational burdens of microbiota comparisons. Thus, we have developed a simple, rapid, and accurate method, independent of multiple alignments and phylogenetic inference, to support microbiota comparisons.

**Results:**

We create a metric, called compression-based distance (CBD) for quantifying the degree of similarity between microbial communities. CBD uses the repetitive nature of hypervariable tag datasets and well-established compression algorithms to approximate the total information shared between two datasets. Three published microbiota datasets were used as test cases for CBD as an applicable tool. Our study revealed that CBD recaptured 100% of the statistically significant conclusions reported in the previous studies, while achieving a decrease in computational time required when compared to similar tools without expert user intervention.

**Conclusion:**

CBD provides a simple, rapid, and accurate method for assessing distances between gastrointestinal tract microbiota 16S hypervariable tag datasets.

## Background

Human-associated microbes outnumber human cells by a factor of ten [1]. The majority of these microbes are harbored in the gastrointestinal tract (GIT) and play a strong role in determining an individual’s health [2]. Commensal GIT microbes may modulate nutrient uptake and utilization, promote GIT development and maturation, extract energy from indigestible non-starch polysaccharides, maintain a healthy immune system, and regulate brain development and behavior [3-5]. Many diseases, ranging from neurological disorders, such as Parkinson’s disease [6], to GIT-related diseases, such as Crohn’s disease (CD) [7], ulcerative colitis (UC) [8], irritable bowel syndrome [9] and obesity [10,11], are correlated with disturbed microbiotas that differ from those of healthy individuals according to some studies. Surveying the microbial diversity in the GIT of patients diagnosed with CD and UC found differing levels of microbial diversity between healthy and diseased GIT samples [7,8]. Evidence examining GIT from obese humans and mice exhibited a markedly decreased fraction of *Bacteroides* and a remarkably increased fraction of *Firmicutes*[10,11]. These studies suggest a strong link between GIT microbial composition and the GIT-related diseases. Recent work has correlated the alleviation of disease symptoms with treatments that alter the microbiota such as fecal transplants [12]. For example, recurrent *Clostridium difficile*-associated infections have been treated using fecal microbiome transplantation (FMT) [13]. The study showed that after two weeks, patient prognosis vastly improved and correspondingly, the fecal bacteria composition of the patient became similar to that of the healthy donor [12]. While many of these results are preliminary [14-16] in nature, they all point to an area of rich research and the growing importance of the GIT microbiota.

The GIT microbiota composition has profound health implications. Modern characterization of GIT microbes is based on culture-independent methods using 16S ribosomal RNA gene (rDNA) hypervariable tag sequencing technologies [17]. 16S rDNA is the most widely used marker for microbial species identification [18]. Currently, next-generation 16S rDNA-based sequencing produces millions of sequences from single run. This advance in sequencing technologies, however, represents a significant methodological challenge. Widely used methodologies include LIBSHUFF [19,20], analysis of molecular variance (AMOVA) [21-23], parsimony tests [23-25] and UniFrac [26-28]. LIBSHUFF uses the Cramer-von Mises statistic to assess whether or not two microbial communities have the same structure [19,20]. AMOVA determines whether or not there is a significant difference between the diversity within the two populations and the diversity of all the populations pooled [21-23]. Parsimony tests describe whether or not two community structures significantly differ from each other [23-25]. UniFrac uses phylogenetic information to detect differences between two microbiotas [26-28]. One weakness of the above methods is that they rely on multiple alignments and/or phylogenetic inference, making them time consuming and requiring exceptional expertise and computational resources. Small changes in algorithms and parameters can have significant influences on the results of microbiota comparisons [29-31]. The issue created by multiple alignments and phylogenetic inference is the rapid growth of the search space for identifying the optimal multiple alignments and phylogenetic trees with the number of sequences [32]. As the ability to sequence continues to outpace advances in computer hardware, more efficient computational algorithms with little or no sacrifice to accuracy will become necessary.

Data-compression techniques based on the notion of Kolmogorov complexity provide an alternative for microbiota comparisons that bypasses multiple alignments and phylogenetic inference. Kolmogorov complexity is defined as the minimum amount of information to reproduce a set of data [33]. As such, Kolmogorov complexity serves as a measure of the repetitiveness within a data set—a powerful proxy for measuring the similarities and differences between datasets [34-36]. However, this theoretically defined concept cannot be computed exactly. Instead, compression algorithms are often used as an approximation for the Kolmogorov complexity [34,35]. The idea of using compression-based metrics on biological data has a long and established history. Data-compression techniques have been used to construct phylogenetic trees [37], analyze mitochondrial genomes [35], classify protein sequences [38], quantify the time-evolution of macrophage gene expression [36], and classify 16S rDNA sequences at family level [39]. Here, we extend the application of a data-compression method for microbiota comparisons based on the repetitive nature of 16S rDNA hypervariable tag sequencing.

**Figure 1:**
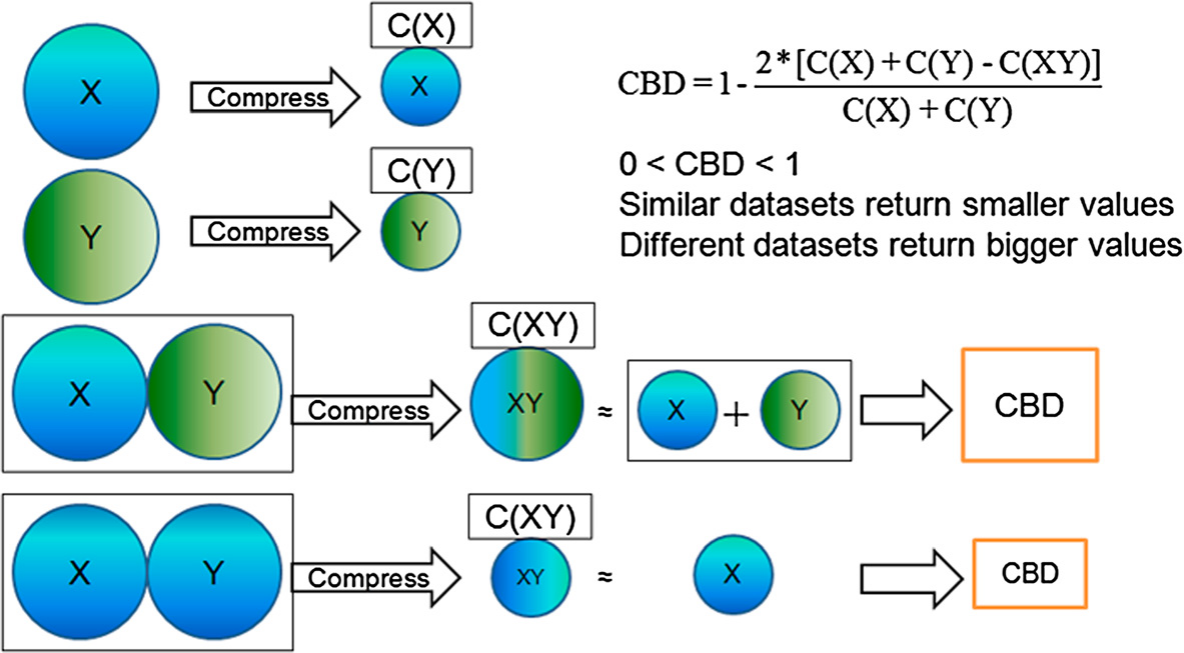
**Schematic of how CBD measures distance between two microbiotas.** Two microbial communities (denoted X and Y) have been characterized using 16S variable tag sequencing. C(X), C(Y), and C(XY) denote the compressed sizes of dataset X, dataset Y, and the concatenation of datasets X and Y. The less similarity between X and Y, the less compression their concatenated dataset, XY, undergoes. In the limit of completely different microbiomes, the size of the compressed dataset C(XY) is equal to the sum of each compressed dataset, C(X) + C(Y). Conversely, when datasets X and Y are very similar, C(XY) is smaller than C(X) + C(Y), leading to a smaller CBD value.

In order to efficiently assess differences in GIT microbiota compositions, we develop a simple, rapid, and accurate method called compression-based distance (CBD) to quantitatively analyze similarities between microbiota samples. As shown in Figure 1, we characterize the similarities between microbial communities via the amount of repetition or overlap in order to determine microbial community distance. CBD relies on the fact that the more repetitive data is the more it can be compressed. By combining 16S rRNA hypervariable tag data from different samples and assessing the relative amounts of compression, we gain a proxy for the similarities between the communities. We convert this to a distance with a minimum of 0 and a maximum of 1 by taking compression gained by combining the datasets over the total compressed size of the individual datasets.

One advantage of CBD is that it operates more directly on the quality-filtered sequence data to generate distance matrices, thus omitting the need for expert intervention in multiple alignments and phylogenetic inference. In this study, three previously published GIT microbiota datasets were used to demonstrate simplicity, speed and accuracy in the application of CBD on GIT microbiotas comparisons. Although compression algorithms can be parameterized to achieve different levels of compression, our applications of these algorithms were done without any significant parameter tuning, highlighting an important practical advantage of CBD.

## Results

CBD provides a one-shot method for determining the level of similarities between two microbiotas. CBD omits the need for expert interventions in assigning similar sequences to OTUs as well as aligning sequence reads, generating phylogenetic trees, realigning sequence reads, and choosing proper software and parameters. For comparison purposes, we used the microbiota analysis toolboxes mothur and QIIME which have implemented automated to semi-automated functions for microbiota comparisons such as UniFrac (Table 1) [40,41].

**Table 1:** Comparisons of CBD with mothur and QIIME

	**Mothur**	**QIIME**	**CBD**
Interface	Command line	Command line	Web or command line
OTUs	Yes	Yes	No
Alignment	Yes	Yes	No
Phylogenetic tree	Yes	Yes	No
Software	Yes	Yes	No
Parameters	Yes	Yes	No

“Interface” indicates how a user communicates with a computer; “OTUs” indicates that clustering methods or algorithms, such as cd-hit, BLAST, furthest neighbor and nearest neighbor, must assign similar sequences to operational taxonomic units (OTUs) to generate a distance matrix; “Alignment” indicates methods such as PyNAST, MUSCLE, and SILVA required to align sequences for generating a distance matrix; “Phylogenetic tree” indicates methods such as FastTree are needed to produce a phylogenetic tree to generate a distance matrix; “Software” indicates a user needs to select different methods or algorithms for choosing OTUs, align sequences and build a phylogenetic tree; “Parameters” indicates a user must choose different parameters associated with a corresponding software.

**Figure 2:**
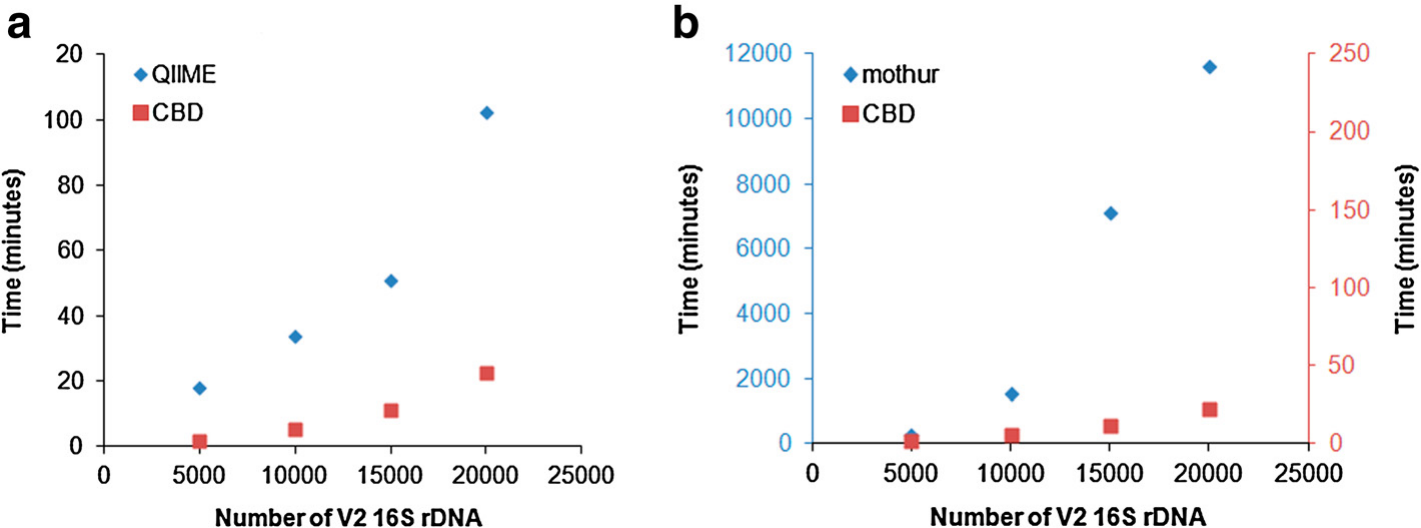
**Speed comparisons between CBD, QIIME and mothur using GIT microbiota of lean and obese twins**[42]**.** Computational time for CBD versus (**a**) QIIME and (**b**) mothur were compared for a variety of dataset sizes. CBD was faster for all datasets tested, particularly for comparisons between large datasets.

We test the computational efficiency of CBD and find it to be dramatically faster for tested sizes of sequences than the popular alternative microbiota comparisons methods, e.g., QIIME and mothur, which are also capable to taking an input dataset and outputting a microbial comparison in an automated fashion (see Figure 2). Furthermore, the advantages of CBD grow as the size of the input files increased.

In order to assess the accuracy of CBD, three published datasets were chosen to repeat previous analyses using distances obtained from CBD: (1) human GIT microbiota [42]; (2) humanized mouse GIT microbiota [43]; and (3) human mucosa-associated microbiota [44].

### Human GIT microbiota

Turnbaugh *et al.*[42] used unweighted UniFrac to analyze a total of 1,937,461 V2 and V6 bacterial 16S rDNA sequences from fecal samples of 154 individuals (31 monozygotic, 23 dizygotic twin pairs, and their mothers). The average sequences per V2 and V6 sample were 3,984 ± 232 and 24,786 ± 1,403, respectively. This revealed that family members had greater similarity in their GIT microbiota composition than unrelated individuals; there is a much greater resemblance in the GIT microbiotas of lean or obese related individuals than lean or obese unrelated individuals [42]. The data were then reanalyzed and compared with previously published results.

**Figure 3:**
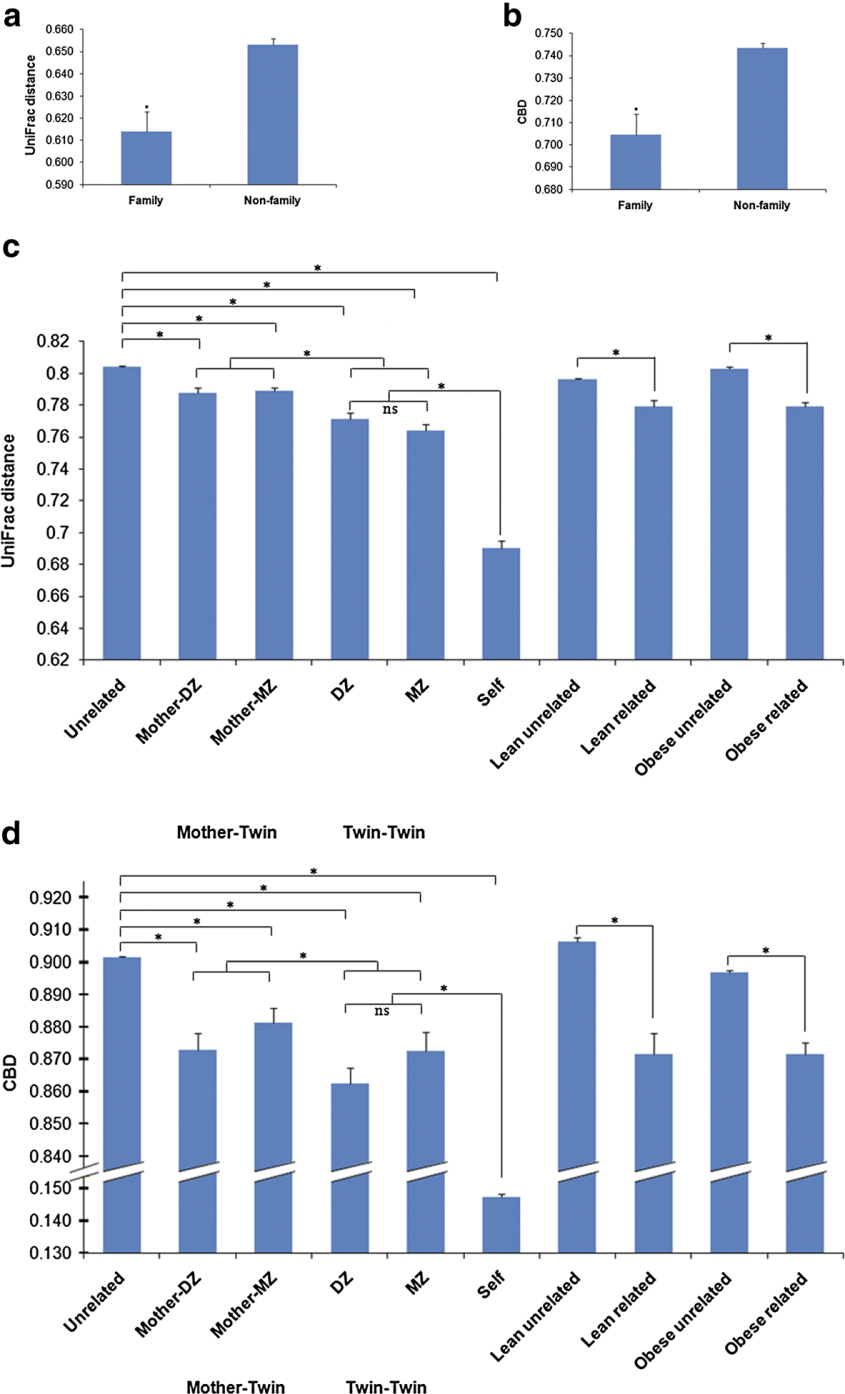
**Comparison of 16S rDNA UniFrac and CBD using GIT microbiota of lean and obese twins**[42]**.** CBD run on V6 and V2 16S rDNA sequences (average 24,786 ± 1,403 sequences per V6 sample and average 3,984 ± 232 sequences per V2 sample) demonstrated agreement with UniFrac analysis [42]. (**a**) Average unweighted UniFrac distance between family and non-family from Turnbaugh *et al.*[42] The graph was reproduced according to the value in the y-axis of Supplemental figure 1a from Turnbaugh *et al.*[42] (**b**) Average CBD between family and non-family (* P < 0.001; mean ± SEM). (**c**) Average unweighted UniFrac distance between Twin-Twin, Mother-Twin, and family-unrelated individuals, lean related and lean unrelated individuals, obese related and obese unrelated individuals from Turnbaugh *et al.*[42]. The graph was reproduced according to the value in the y-axis of Figure 1a from Turnbaugh *et al.*[42] (**d**) Average CBD between Twin-Twin, Mother-Twin, and family-unrelated individuals, lean related and lean unrelated individuals, obese related and obese unrelated individuals (* P < 0.05; mean ± SEM).

For consistency, we only consider comparisons that resulted in statistically significant differences between groups. Comparisons using CBD analysis on V6 16S rDNA sequences between family and non-family were consistent with the analysis using UniFrac (Additional file 1: Table S1, Figure 3a and 3b) [42]. Analyses on V6 16S rDNA datasets showed that CBD recaptured the conclusions from previous analyses. However, V6 16S rDNA datasets averaged about 24,000 reads, whereas clinically, one would prefer to utilize shallower sampling of the GIT microbiota for cost efficiency with the same confidence level. V2 16S rDNA datasets had an average of about 4,000 reads and were used to test the performance of CBD under a restricted information circumstance. Comparisons using CBD analysis on V2 16S rDNA sequences were consistent with the analysis using UniFrac (Additional file 1: Table S1, Figure 3c and 3d) [42]. Analyses on V2 16S rDNA datasets revealed that CBD performed well as UniFrac on computing similarities among multiple microbiota categories. CBD using 16S rDNA sequences provided sixteen out of sixteen distance relationships matching those found by Turnbaugh *et al.* (Additional file 1: Table S1, Figure 3) [42].

### Humanized mouse GIT microbiota

**Figure 4:**
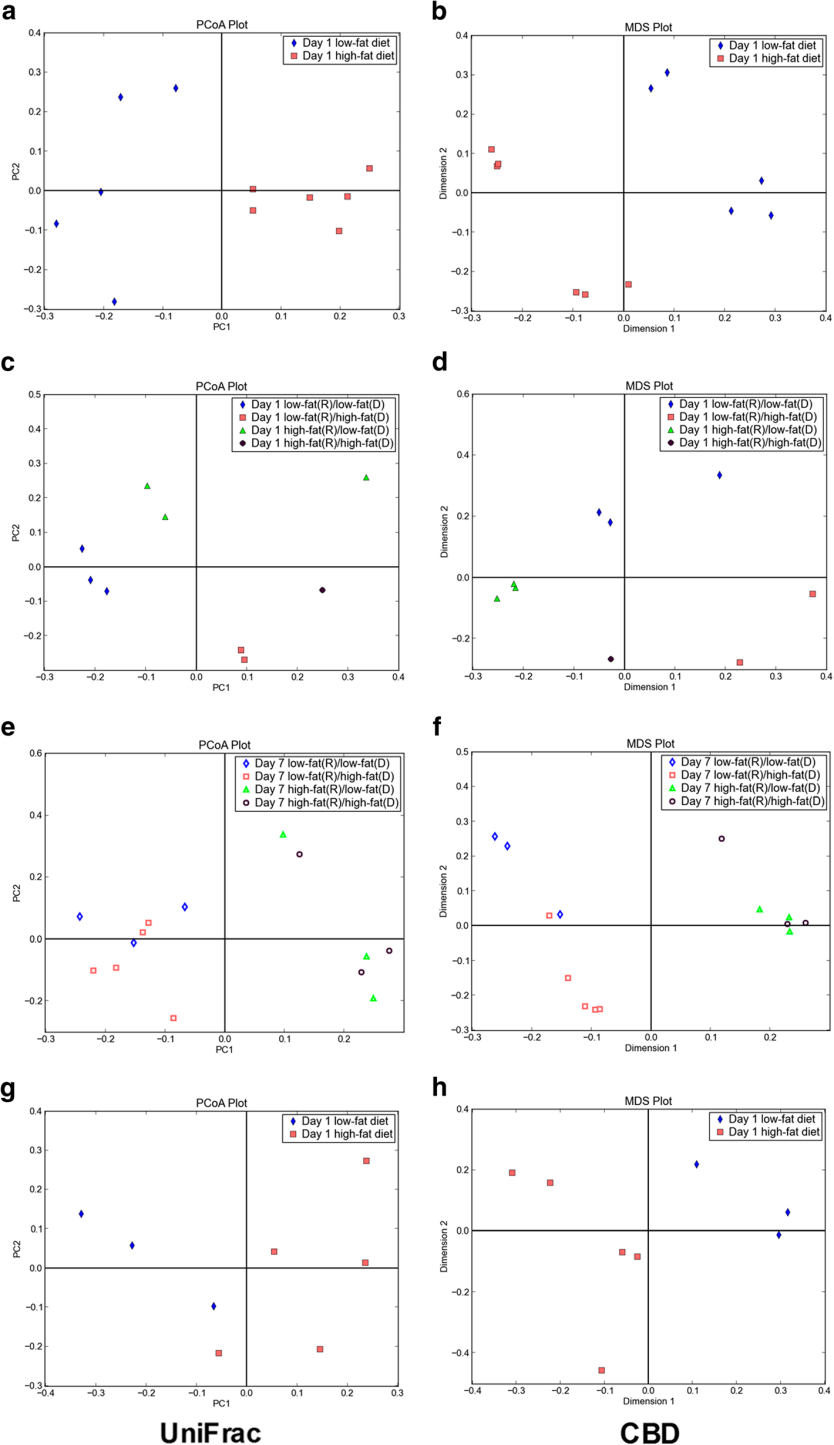
**Comparison of 16S rDNA UniFrac and CBD using humanized mouse GIT microbiota**[43]**.** CBD analyses using V2 16S rDNA sequences were consistent with the UniFrac analyses [43]. (**a** or **b**) UniFrac-based principal component plots (PCoA) reproduced based on previously published analysis and CBD-based multidimensional scaling analysis (MDS) showed clustering of microbiotas by diet. The microbiotas were collected from mice transferred fresh human feces on first day after diet switch. (**c** or **d** or **e** or **f**) UniFrac-based PCoA reproduced based on previously published analysis and CBD-based MDS revealed that mice fed low-fat diet but with microbiota from humanized mice fed high-fat diet and mice fed high-fat diet but with microbiota from humanized mice fed low-fat diet showed intermediate clustering on day 1 while clustered in accordance with recipient diet on day 7. (**g** or **h**) UniFrac-based PCoA reproduced based on previously published analysis and CBD-based MDS showed clustering of microbiotas by diet. The microbiotas were collected from mice transferred frozen human feces on first day after diet switch. The UniFrac distance matrix, which was used to produce Figure 4a, 4c, 4e, and 4g, was generated by QIIME with default parameters (except using cd-hit for OTUs picking) from V2 16S rDNA sequences downloaded from Turnbaugh *et al.*[43]. In Figure 4a, 4b, 4g, and 4h, blue diamonds and red squares indicate samples collected from mice fed low-fat and high-fat diet, respectively. In Figure 4c and 4d, blue diamonds indicate samples collected from low-fat donor and low-fat recipient on first day; red squares indicate samples collected from high-fat donor and low-fat recipient on first day; green triangles indicate samples collected from low-fat donor and high-fat recipient on first day; purple circles indicate samples collected from high-fat donor and high-fat recipient on first day. In Figure 4e and 4f, corresponding hollow patterns represent samples collected on day 7.

Turnbaugh *et al.*[43] used unweighted UniFrac to analyze V2 16S rDNA sequence data to investigate the effect of diet on humanized murine GIT microbiota composition. They transferred fresh or frozen human feces into germ-free mice and observed the effect of a dietary switch from low-fat to high-fat diet on humanized mouse GIT microbiota. They also transferred microbiota from humanized mice fed low-fat or high-fat diet into germ-free mice to observe the effect of the diet switch from low-fat to high-fat diet on humanized mice. They revealed that the dietary switch induced changes in the composition of humanized GIT microbiota within one day. Samples taken from mice on a low-fat diet with transplanted microbiota from mice on high-fat diets and mice on a high-fat diet with transplanted micro biota from mice on low-fat diets showed intermediate clustering by day 1 while clustered in accordance with recipient diet by day 7. The V2 16S rDNA sequence data were reanalyzed using CBD to determine the impact of diet manipulation on humanized GIT microbiota composition. CBD analyses of V2 16S rDNA sequences were consistent with those analyses using UniFrac (Figure 4) [43].

### Human mucosa-associated microbiota

**Figure 5:**
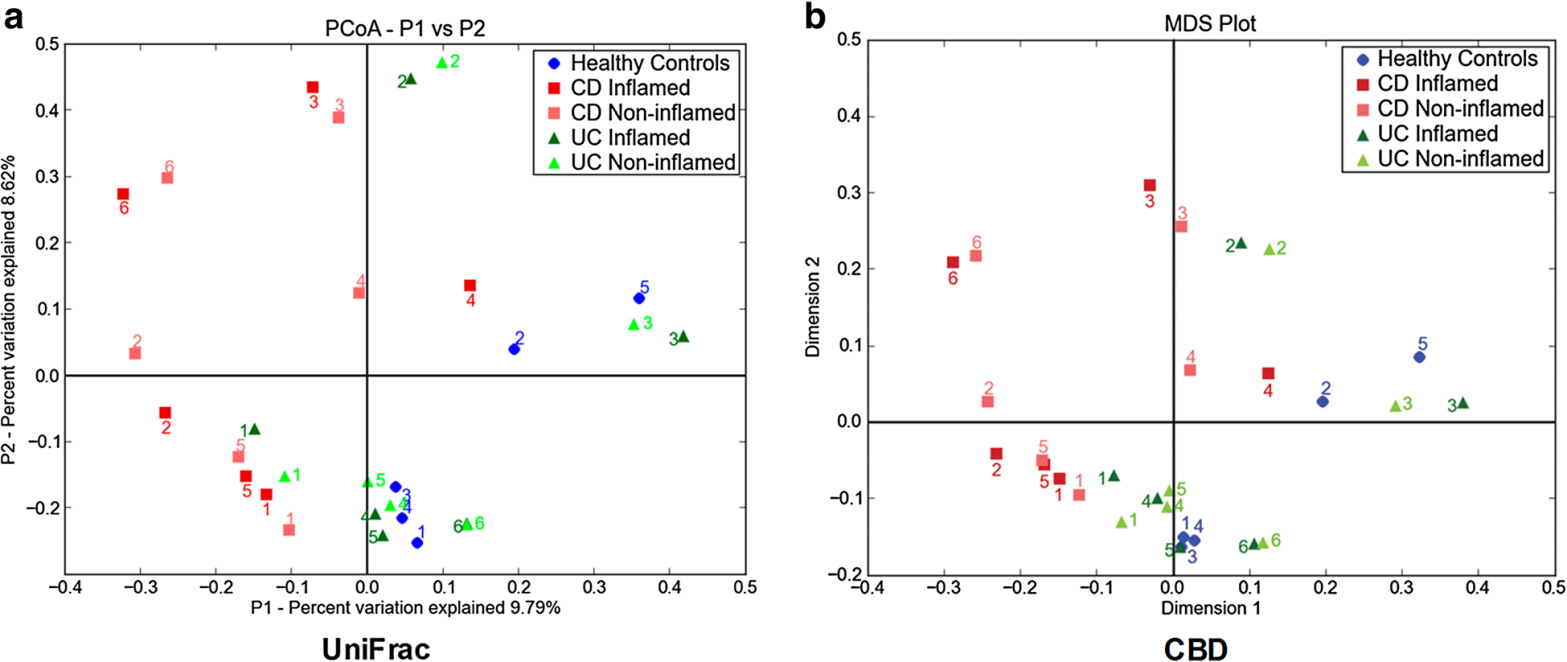
**Comparison of 16S rDNA UniFrac and CBD using human mucosa-associated microbiota**[44]**.** CBD analyses using full-length 16S rDNA sequences were consistent with the Fast-UniFrac analyses [44]. (**a**) Clustering of individual microbiotas using UniFrac-based PCoA. The graph was reproduced according to the values in the Figure 5 from Walker *et al.*[44]. (**b**) Clustering of individual microbiotas using CBD-based MDS. Each dot represents an individual sample. Blue circles indicate healthy controls. Red squares donate CD patients. Green triangles represent UC patients.

Walker *et al.*[44] determined the effects of disease on human GIT microbiota compositions. Full-length mucosa-associated bacterial 16S rDNA from inflamed and non-inflamed regions of 6 UC and 6 CD patients were compared to those from 5 healthy controls. Their study revealed that mucosa-associated microbiotas clustered as individuals rather than by disease cohort. CBD was used to reanalyze the data to reveal the relationships between diseased and healthy GIT microbiotas. The CBD analyses using full-length 16S rDNA sequences were consistent with the analysis using UniFrac (Figure 5) [44].

## Discussion

The development of advanced and cost-effective DNA sequencing techniques enables the generation of tremendous datasets. For example, three recent studies reported that Illumina GAIIx or HiSeq platform produced millions of reads [45-47]. To accommodate this high-throughput data generation, simple and fast tools are extremely important for efficiently and accurately extracting information to further characterize microbiota. Increasing the efficiency of microbial community comparisons has profound implications for research. The CBD method described here facilitates efficient similarity comparisons between microbiotas.

CBD generates the distance matrix directly from sample sequences in relatively few steps. In contrast, the tree-based metric required multiple steps including assignment of OTUs, alignment, production of phylogenetic trees and generation of a distance matrix [42]. Furthermore, Caporaso *et al.*[41] determined that approximately 92% of the computational time was devoted to picking OTUs rather than determining distance assessment. Compared to QIIME and mothur, CBD required much less time completing the distance matrix from large numbers of sequences.

The accuracy of CBD was demonstrated by the reproduction of the statistical relationships between different classes of microbiotas and the ability to reproduce the results from microbial comparison using various methods. In this way, CBD was shown to be a robust and useful tool. However, we note that CBD is not a wholesale replacement for more involved analyses. For example, CBD does not provide information such as taxa or OTU distributions. It provides a simple, rapid, and accurate metric for comparing distances between entire communities of microbes, not a fine-grained assessment of particular species within a community.

The simplicity, speed, and accuracy of CBD suggests that it facilitates microbiota research when used in human-related samples. It does not require enormous sequencing depths obtained from non-invasively collected stool samples, and it is relatively simple for a biological/clinical researcher to compute CBD values. There is increasing evidence advancing the application of GIT microbiota assessments. Smith *et al.*[48] have implicated the GIT microbial composition as a causal factor of Kwashiorkor. Qin *et al.*[49] reported that the GIT microbiota of CD patients could be differentiated from that of healthy controls and UC patients based on the abundance of 155 bacterial species. Khoruts *et al.*[12] observed two weeks after fecal transplantation that fecal microbes of *Clostridium difficile*-associated disease patients were similar to those of healthy donors. In a recent study, switching mice from a low-fat diet to a high-fat diet was shown to abruptly change the population of GIT microorganisms within one day [43]. Potentially, CBD could aid more informed microbial management by comparing the microbiota before, during, and after manipulation. It could facilitate the exploration of new treatment strategies, and it could be used for diagnosis and prognosis of GIT-related diseases.

The focus of this work was to explore CBD as a tool for microbiota community comparison with a focus on clinical applications. However, the principles behind CBD should be equally applicable to any set of sequenced amplicons. This may be useful in other studies related to the microbiota that focus on fungal or other eukaryotic organisms in the gastrointestinal tract or other environments by examining 18S rRNA hypervariable tag sequencing or internal transcribed spacer regions (ITS).

CBD is web-based and freely accessible at http://tornado.igb.uiuc.edu/CBD/CBD.html. Sequence data in FASTA format can be directly uploaded to the CBD website for analysis. CBD is copyrighted by the board of trustees of the University of Illinois.

## Conclusion

CBD provides a simple, rapid but accurate method for microbiota comparisons. It uses the relative compression of combined and individual datasets to quantify overlaps between two microbial communities, therefore is independent of multiple alignments and phylogenetic inference. CBD worked directly on sequence datasets without intermediate steps. The speed advantages of CBD over pipelines in QIIME and mothur became more pronounced as dataset size increased. Tests run on previously analyzed data indicated strong agreement between CBD and more time-consuming analyses.

## Methods

### Compression-based distance

We developed a new method, Compression-based Distance, to assess similarities between two 16S amplicon datasets, X and Y. CBD uses the relative compression of the concatenated 16S rDNA hypervariable tag sequencing datasets XY and individual 16S rDNA hypervariable tag sequencing datasets X and Y to produce a distance value to quantify overlaps between two microbial communities according to the following formula:

$$\mathrm{CBD}=1-2*\frac{C\left(X\right)+C\left(Y\right)-C\left(\mathrm{XY}\right)}{C\left(X\right)+C\left(Y\right)}$$

where C(X) indicated the size of data X after compression, C(Y) indicated the size of data Y after compression, and C(XY) denoted the size of concatenated data XY, where data Y was concatenated to the end of data X, again after compression. Lempel-Ziv-Markov chain-Algorithm (LZMA) compressor (compression level −9) was used. The range of scores from CBD was between 0 and 1 (0, 1) with similar datasets returning smaller values and different datasets returning greater values. The similarity between two microbiota calculated by CBD metric was influenced by two factors, the number of similar sequences between two microbiota and total size of the concatenation of two microbiota datasets. For the same number of similar sequences, the bigger the total size of the concatenation of two microbiota datasets, the greater the CBD value was.

The specific tool we chose for compress (LZMA) was based on tests that indicated LZMA provided better compression ratios in comparison to other commonly available compression tools such as zip, gzip, or bz2. For all datasets, we removed the sequence labels before compressing so that the sequence names do not affect our results. Our datasets were then sorted before compression in order to improve the compression ratio further. Sorting resulted in a large performance boost, especially for larger datasets that were larger than the memory footprint of the compression algorithm, by placing similar sequences near each other in memory.

### Test of CBD on artificial datasets

**Figure 6:**
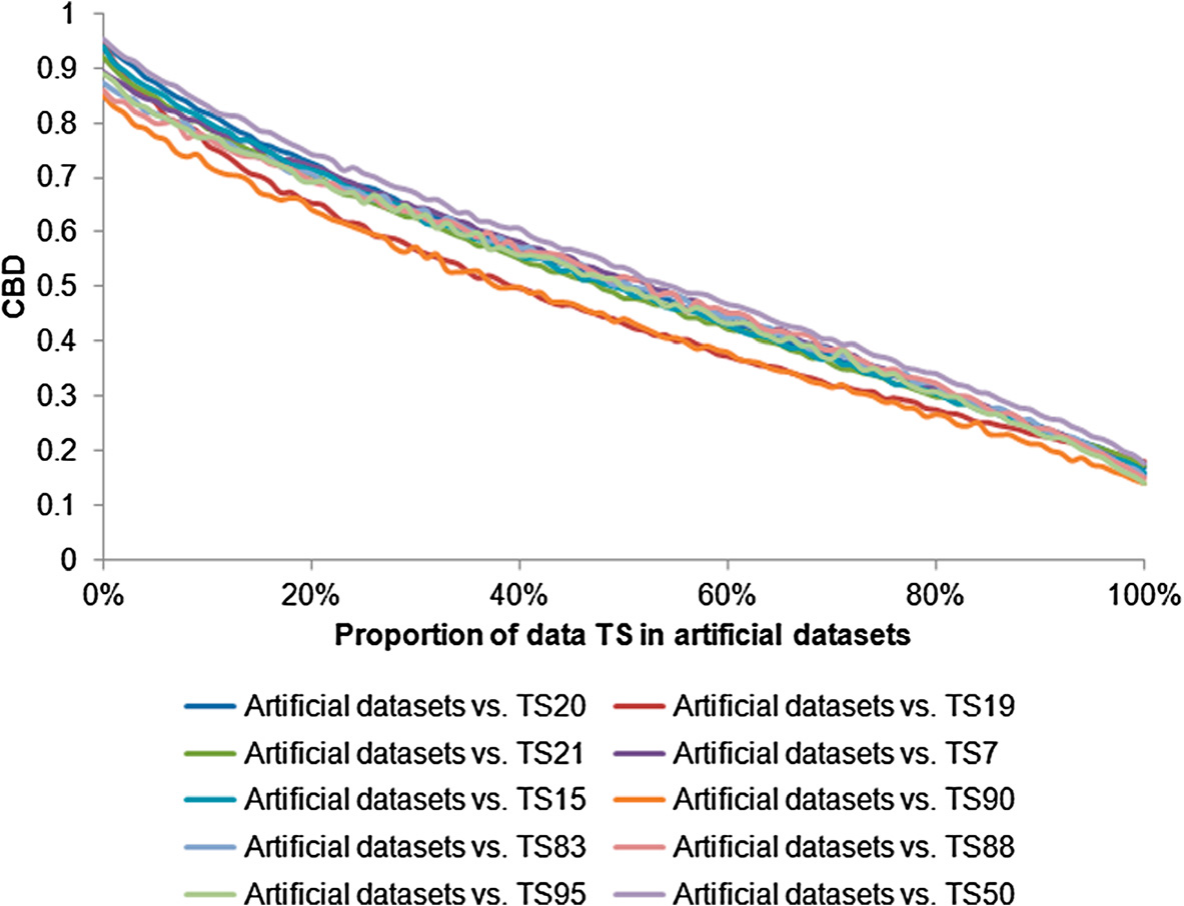
**CBD values for individual pairwise comparisons between artificial datasets and TS dataset.** TS dataset represents one of the following: TS20_V2 data, TS19_V2 data, TS21_V2 data, TS7_V2 data, TS15_V2 data, TS90_V2 data, TS83_V2 data, TS88_V2 data, TS95_V2 data, and TS50_V2 data. Artificial datasets were constructed from mixtures of TS20_V2 and TS51_V2 dataset, TS12_V2 and TS19_V2 dataset, TS9_V2 and TS21_V2 dataset, TS7_V2 and TS27_V2 dataset, TS15_V2 and TS30_V2 dataset, TS90_V2 and TS91_V2 dataset, TS74_V2 and TS83_V2 dataset, TS88_V2 and TS103_V2 dataset, TS95_V2 and TS104_V2 dataset, TS50_V2 and TS64_V2 dataset, respectively. CBD value decreased with increased proportion of TS dataset in artificial datasets as expected.

A reliable metric of community distance will return greater values for communities that were more distant and smaller values for communities that were virtually the same. In order to test if CBD met these criteria, we applied CBD to ten sets of artificial datasets generated by sampling different proportions of sequences obtained from ten different pairs of individuals (merged TS20_V2 and TS51_V2 data, merged TS12_V2 and TS19_V2 data, merged TS9_V2 and TS21_V2 data, merged TS7_V2 and TS27_V2 data, merged TS15_V2 and TS30_V2 data, merged TS90_V2 and TS91_V2 data, merged TS74_V2 and TS83_V2 data, merged TS88_V2 and TS103_V2 data, merged TS95_V2 and TS104_V2 data, merged TS50_V2 and TS64_V2 data from a recent GIT microbiota study of obese and lean twins) [42]. This allowed us to know, *a priori*, the relative similarities between the datasets. In Figure 6, CBD for each pairwise comparison between the artificial datasets and TS20_V2 data or TS19_V2 data or TS21_V2 data or TS7_V2 data or TS15_V2 data or TS90_V2 data or TS83_V2 data or TS88_V2 data or TS95_V2 data or TS50_V2 data were plotted. As expected, CBD revealed that distances decrease with an increasing proportion of sample overlap, verifying that CBD reliably assessed similarities. Furthermore, the response function appeared to be nearly linear and utilized almost the full range of values from 0 to 1. This suggested that the metric had the appropriate scale of sensitivities for GIT defined datasets. Artificial datasets and distance matrices can be freely downloaded at http://tornado.igb.uiuc.edu/CBD/CBDFiles/CBDDownload.html.

**Figure 7:**
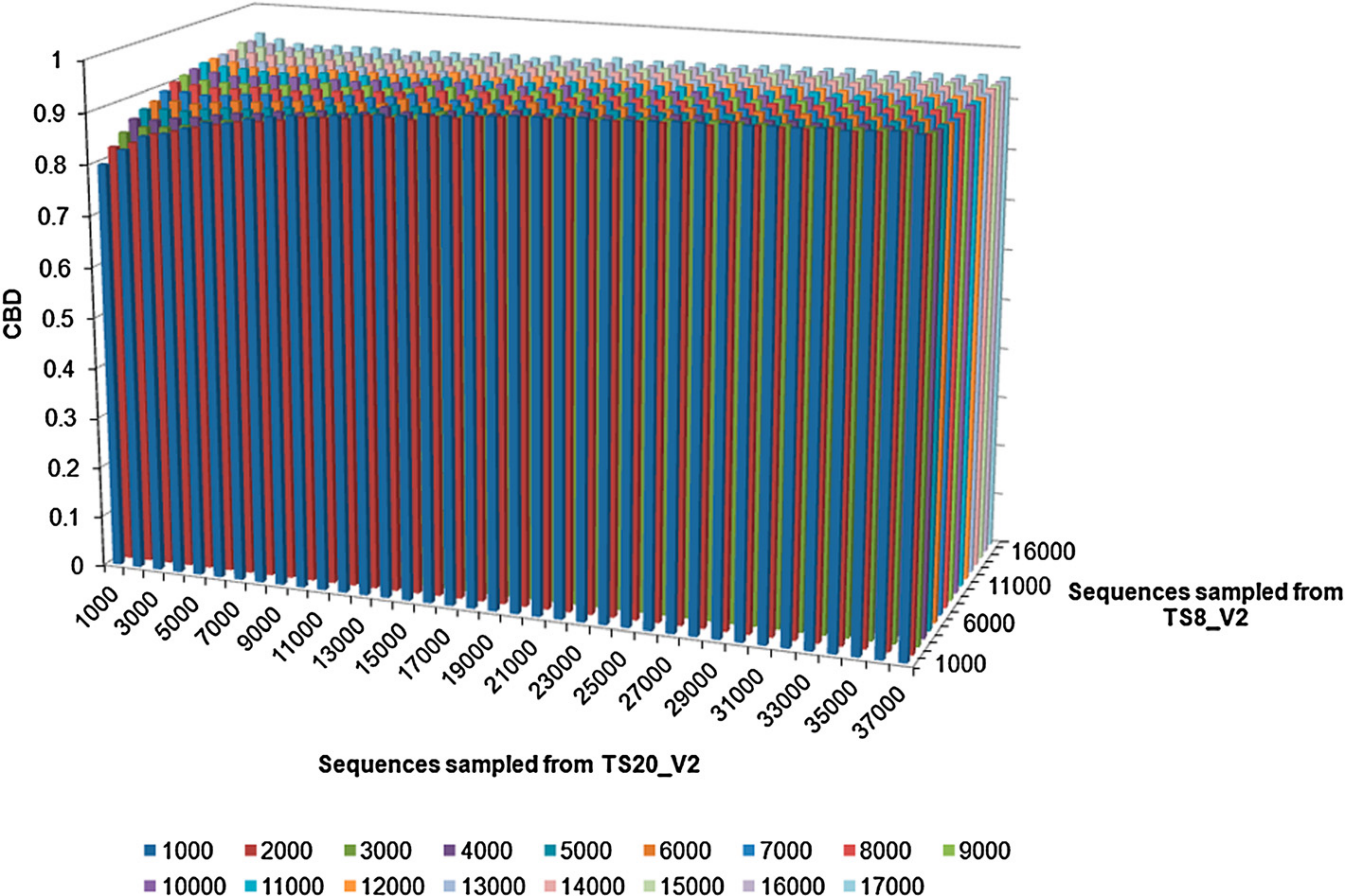
**CBD values for individual pairwise comparisons between artificial datasets.** CBD values for individual pairwise comparisons using artificial datasets produced by randomly sampling different numbers of sequences from TS8_V2 and TS20_V2 dataset. CBD metric was not very sensitive to absolute size except for extremely small values.

We have shown CBD to be sensitive to changes in microbiota composition. We now examine it’s robustness to finite size effects from comparisons between datasets of different sizes. As a test, artificial datasets generated by randomly sampling different numbers of sequences obtained from two individuals (merged TS8_V2 and TS20_V2 from a recent GIT microbiota study of obese and lean twins) [42] were pairwise compared with each other to obtain CBD values. TS8_V2 and TS20_V2 contained 17,000 and 37,000 sequences, respectively. In Figure 7, CBD for each pairwise comparison among the artificial datasets were plotted. It revealed that CBD value was primarily a function of the number of overlap between two datasets and not the total size of any particular dataset. The overall CBD value remained largely robust to the size of the datasets past 4000 reads per sample. Artificial datasets and distance matrices can be freely downloaded at http://tornado.igb.uiuc.edu/CBD/CBDFiles/CBDDownload.html.

**Figure 8:**
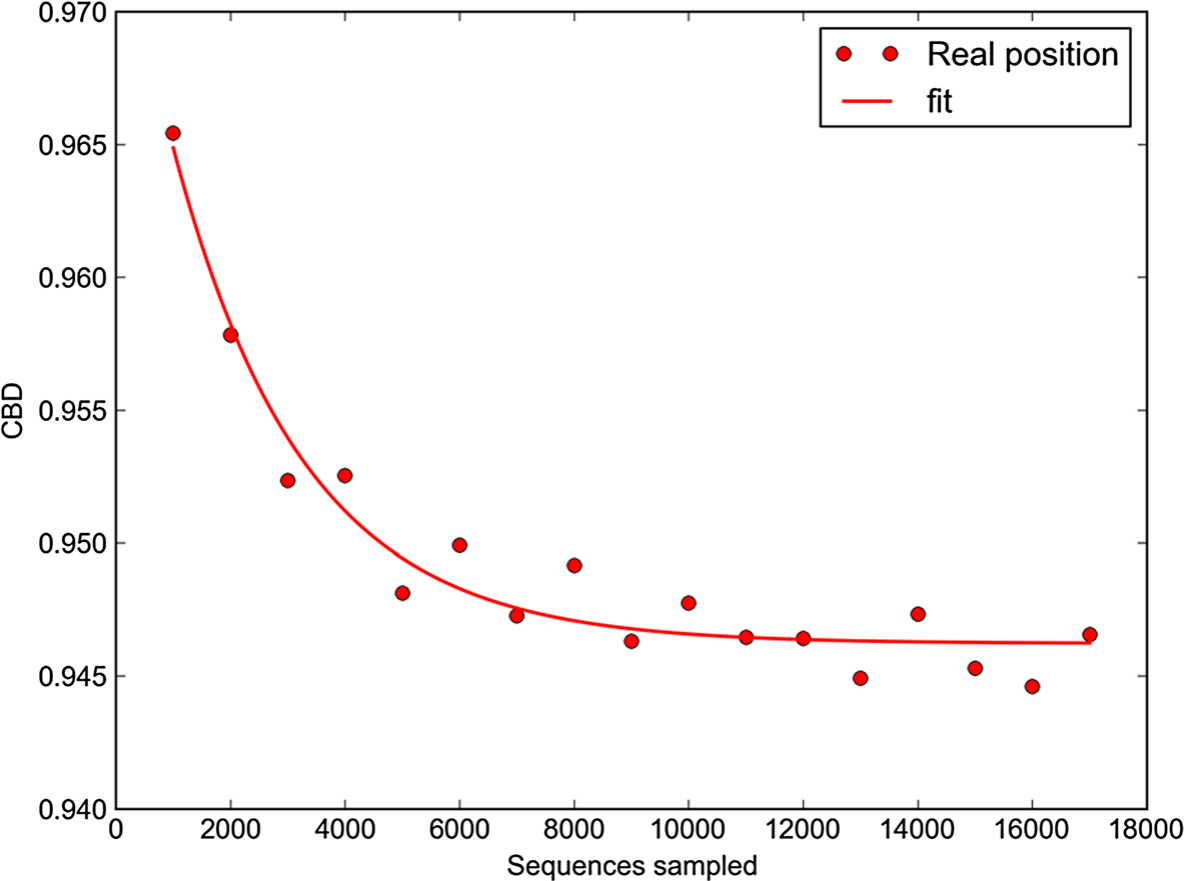
**CBD values for individual pairwise comparisons between artificial datasets and TS20_V2 dataset.** CBD values for individual pairwise comparisons between artificial datasets produced by randomly sampling different numbers of sequences from TS8_V2 and TS20_V2 dataset. CBD metric converges rapidly with sample size. Note that the Y-axis spans a mere 4% of the CBD scale. The scale of the Y-axis has been blown up in this way so that we could display the differences between different points to the reader.

In order to further quantify the influence of sequence library size on CBD value, artificial datasets generated by randomly sampling different numbers of sequences obtained from an individual (merged TS8_V2 from a recent GIT microbiota study of obese and lean twins) [42] were pairwise compared with another individual (merged TS20_V2 from a recent GIT microbiota study of obese and lean twins) [42] to obtain CBD value. In-house python script was used to do an exponential curve fitting. In Figure 8, CBD for each pairwise comparison among the artificial datasets and TS20 dataset were plotted. Figure 8 revealed that CBD metric converges rapidly with sample size. Artificial datasets and distance matrices can be freely downloaded at http://tornado.igb.uiuc.edu/CBD/CBDFiles/CBDDownload.html.

### Datasets used in this analysis

In this study, three previously published GIT microbiota datasets were used: 1) V2 and V6 16S rDNA datasets from a recent study that focused on the GIT microbiotas of lean and obese twin pairs and their mothers [42]; 2) V2 16S rDNA datasets from an analysis of the effect of diet switch from low-fat diet to high-fat diet on humanized murine GIT microbiota composition [43]; and 3) full-length 16S rDNA datasets from mucosa-associated microbiotas from inflamed and non-inflamed sites of CD and UC patients in the colon as well as that from healthy controls [44]. These datasets were used to test if CBD could successfully recapture the conclusions of previous clinical studies. The links to the three published GIT microbiota datasets can be found at http://tornado.igb.uiuc.edu/CBD/CBDFiles/CBDDownload.html. The first human GIT microbiota data was also used to assess the speed of CBD.

### Measurement of computational time

The first five, ten, fifteen, and twenty V2 16S rDNA datasets at the first time point in Additional file 1: Table S1 of Turnbaugh *et al.*[42] were chosen to form four group files. One thousand sequences were randomly chosen from each file within the group files to be pairwise compared to each other using CBD or QIIME pipeline (http://qiime.sourceforge.net) with default parameters (except using cd-hit for OTUs picking) or mothur (using unique.seqs to remove identical sequences, align.seqs to align unique sequences, clearcut to produce neighbor joining trees, and unifrac unweighted to generate UniFrac distance matrix) in order to produce a CBD distance matrix or an unweighted UniFrac distance matrix [40,41]. Because QIIME integrates many 16S rDNA analysis software tools into one system, the fastest way to run QIIME (v.1.2.0) is to build QIIME Virtual Box, which requires at least 1024 MB memory, 120 GB storage and a 64-bit system [41], the time analysis of CBD and QIIME was operated using same computer configuration (8 Intel(R) Xeon(R) CPU E5504 at 2.00 GHz). Because the generation of tree file with clearcut command in mothur v.1.24.1 requires large amounts of memory (RAM), the time analysis of CBD and mothur was operated in large memory cluster located at Institute for Genomic Biology at University of Illinois at Urbana-Champaign (2 Nodes, 16 2.4 GHz Intel CPUs and 256 GB of RAM as well as 24 2.0 GHz Intel CPUs and 1024 GB of RAM) [40,41]. Sequence data used to measure the computational time can be downloaded at http://tornado.igb.uiuc.edu/CBD/CBDFiles/CBDDownload.html.

### Mantel test for dissimilarity between CBD and UniFrac matrix

The Mantel statistic based on Pearson’s product–moment correlation with 1000 permutations was used to evaluate relation between CBD and unweighted UniFrac distance matrix. The first twenty V2 16S rDNA datasets at the first time point in Additional file 1: Table S1 of Turnbaugh *et al.*[42] was used to perform Mantel test in R language (v.2.11.1). Pearson correlation coefficient between CBD matrix and unweighted UniFrac distance matrix obtained from mothur was 0.868 (P-value = 0.001), which suggests that CBD distance matrix and mothur distance matrix were statistically, significantly, highly and positively related to each other. Pearson correlation coefficient between CBD matrix and unweighted UniFrac distance matrix obtained from QIIME was 0.208 (P-value = 0.035). This suggests that there are lesser, but still statistically significant correlation between the CBD distance matrix and the QIIME distance matrix. Pearson correlation coefficient between the mothur distance matrix and the QIIME distance matrix was 0.226 (P-value = 0.027), which suggests that these matrices are similarly, statistically, significantly and positively associated with each other. While all matrices are significantly correlated, there is a disparity in the amount of correlation, particularly in comparisons of QIIME.

### Distance matrix

Sequence datasets from three previous studies were used to generate a respective distance matrix. In the study of identical and fraternal twin pairs and their mothers [42], V2 16S rDNA sequences from the same person at two different time points were merged. Sequences were sorted for each merged V2 and V6 dataset. All pairs of merged V2 or V6 16S rDNA sequences were then compared using the CBD metric. These pairwise distances were used to generate a distance matrix. Twenty-one pairs of samples were analyzed by CBD (Additional file 1: Table S1). In the study of the effect of diet on humanized murine GIT microbiota, all GIT microbiotas under different diets were pairwise compared to each other to generate a distance matrix [43]. In order to study the effect of disease on GIT microbiota composition, all mucosa-associated microbiotas from CD and UC patients’ inflamed and non-inflamed sites and healthy controls were pairwise compared to generate a distance matrix [44]. The distance matrices can be downloaded at http://tornado.igb.uiuc.edu/CBD/CBDFiles/CBDDownload.html.

### P-values

In the study of identical and fraternal twin pairs and their mothers [42], rows and columns of the distance matrix were randomly permutated 1000 times. In order to determine significant difference, the distribution of these results was compared to the actual values.

### Metric dimensional scaling

In order to visualize the distance relationships between data samples from different individuals, metric dimensional scaling (MDS) in R language (v.2.11.1) was used to convert information into low dimensional and easy-to-visualize space where similarities between data points were conserved as much as possible [50]. A two dimensional MDS representation of distance matrices was visualized in a 2D graphics by matplotlib (Python 2D graphics package used for generating publication-quality images) [51].

## Abbreviations

CBD: Compression-based distance; GIT: Gastrointestinal tract; CD: Crohn’s disease; UC: Ulcerative colitis; rDNA: Ribosomal RNA gene; OTUs: Operational taxonomic units; LZMA: Lempel-ziv-markov chain-algorithm; MDS: Metric dimensional scaling; AMOVA: Analysis of molecular variance; PCoA: Principal coordinates analysis.

## Competing interests

The authors declare that they have no competing interests.

## Authors’ contributions

FY, NC, BAW and LBS conceived and designed the project. FY and NC performed the experiments for the paper. FY drafted the manuscript. FY, NC, BAW and LBS revised the manuscript critically. All authors read and approved the final manuscript.

## Supplementary Material

Additional file 1: Table S1 V2 and V6 16S rDNA CBD metric statistics. Click here for file
