# Supplementary material for: Compression-based distance (CBD): a simple, rapid, and accurate method for microbiota composition comparison
Source: BMC Bioinformatics. 2013 Apr 23;14:136. doi: 10.1186/1471-2105-14-136 (PMC3660234; doi:10.1186/1471-2105-14-136)
Supplement: Additional file 1: Table S1 — V2 and V6 16S rDNA CBD metric statistics. [file 1471-2105-14-136-S1.pdf]

Table S1. V2 and V6 16S rDNA CBD metric statistics.

Pairwise comparisons on V2 16S rDNA sequences	Relation predicted by UniFrac	Absolute distance[a]	Relation predicted by CBD
Unrelated vs. Mother-DZ	>	0.0286 ± 0.0052***	>
Unrelated vs. Mother-MZ	>	0.0204 ± 0.0050***	>
Unrelated vs. DZ	>	0.0390 ± 0.0050***	>
Unrelated vs. MZ	>	0.0290 ± 0.0060***	>
Unrelated vs. Self	>	0.7542 ± 0.0012***	>
Mother-DZ vs. Mother-MZ	ns	0.0082 ± 0.0098	ns
Mother-DZ vs. DZ	>	0.0104 ± 0.0098	ns
Mother-DZ vs. MZ	>	0.0004 ± 0.0108	ns
Mother-DZ vs. Self	>	0.7256 ± 0.0060***	>
Mother-MZ vs. DZ	>	0.0186 ± 0.0096**	>
Mother-MZ vs. MZ	>	0.0086 ± 0.0106	ns
Mother-MZ vs. Self	>	0.7338 ± 0.0058***	>
Mother-Twin vs. Twin-Twin	>	0.0090 ± 0.0074*	>
Mother-Twin vs. Self	>	0.7300 ± 0.0044***	>
Twin-Twin vs. Self	>	0.7210 ± 0.0048***	>
DZ vs. MZ	ns	0.0100 ± 0.0106	ns

DZ vs. Self	>	0.7152 ± 0.0058***	>
MZ vs. Self	>	0.7252 ± 0.0068***	>
Lean Unrelated vs. Lean Related	>	0.0348 ± 0.0076***	>
Obese Unrelated vs. Obese Related	>	0.0254 ± 0.0040***	>

**Pairwise comparisons on V6 16S rDNA sequences**	**Relation predicted by UniFrac**	**Absolute distance[a]**	**Relation predicted by CBD**
Non-family vs. Family	>	0.0390 ± 0.0112***	>

[a]Absolute distance expressed as average absolute distance ± SEM computed by CBD between the two pairs of communities

***, ** and * indicate that the probability of finding an absolute distance number more differentiated than the actual values after random permutation of the rows and columns for 1000 times is less than 0.001, 0.01 and 0.05, respectively

">" indicates that latter pair is more related to each other when compared to previous pair

"<" indicates that previous pair is more related to each other when compared to latter pair

“ns” indicates that the probability of finding an absolute distance number more differentiated than the actual values after random permutation of the rows and columns for 1000 times is greater than 0.05

Note: for consistency, all comparisons involving non-significant cases where there was insufficient data to discern a clear relationship according to one or both of the methods were discarded.
